# The Trauma-Informed Attitudes and Practices of Early Childhood Special Education Teachers: A Mixed Methods Exploration

**DOI:** 10.1007/s40653-026-00818-1

**Published:** 2026-02-12

**Authors:** Mia Chudzik, Catherine Corr, Abby Hardy

**Affiliations:** 1 Department of Special Education, Department of Curriculum & Instruction, University of Illinois, Urbana, Champaign, USA; 2 Department of Special Education, University of Illinois, Urbana, Champaign, USA

**Keywords:** Preschool, Teachers, Trauma, Trauma-informed care

## Abstract

Young children with disabilities experience trauma at high rates. Early childhood special education teachers play a critical role in supporting these children through the use of trauma-informed care. This study explored early childhood special education teachers’ trauma-informed attitudes and practices. Twelve early childhood special education teachers in one Midwestern state participated in this study. A mixed methods study was conducted, with participants completing a survey to measure trauma-informed attitudes and participating in interviews to explore trauma-informed practices. Participants were aware of the impact of trauma on the children in their class and described various factors that impacted their use of trauma-informed care. Implications for research and practice are discussed related to the provision of trauma-informed care for young children with disabilities.

Trauma, including child abuse and neglect, is common in the early childhood years. Estimates suggest that by age five, 55% of children have experienced a traumatic event (Jimenez et al., 2016). Additionally, children under age six experience abuse and neglect at disproportionately higher rates than older children and make up two-thirds of children referred to child welfare for suspected abuse and neglect (U.S. Department of Health and Human Services [HHS], 2024). Young children who have experienced trauma experience a variety of emotional, behavioral, and academic impacts. Additionally, children with disabilities are more likely to experience trauma than children without disabilities (Vanderminden et al., 2023). This can be attributed, in part, to additional stress that their caregivers face due to systemic failures to support families of young children with disabilities properly (Legano et al., 2021; Murphy, 2011; Peer & Hillman, 2014).

The age of birth to five is a critical period due to the rapid pace of brain development (DeBellis & Zisk, 2014; Shonkoff et al., 2009). Trauma that occurs during this time can lead to difficulties with executive functioning (Murray et al., 2015; Op den Kelder et al., 2018), building relationships with peers and adults (Cook et al., 2005; Van der Kolk et al., 2005), emotional identification and regulation (Lubit et al., 2003; Schore et al., 2002), and difficulties with learning (Panlilio et al., 2019). Given the high rates of trauma in young children with disabilities and the significant impact of trauma in early childhood, early childhood education settings are increasingly seen as a place where trauma-informed approaches need to be implemented (Bartlett & Smith, 2019; Sun et al., 2024).

Teachers play a critical role in supporting children who have experienced trauma (Harris, 2018; National Child Traumatic Stress Network, 2017). One way teachers can support this population of children is through trauma-informed care, which has been shown to benefit both young children (Sun et al., 2024) and teachers (Christian-Brandt et al., 2020). Despite the importance of trauma-informed care, little attention has been paid to early childhood special education (ECSE) teachers and their experiences supporting children who have experienced trauma through the use of trauma-informed care (Chudzik et al., 2023).

## Trauma-Informed Care

Trauma-informed care is a service delivery model that focuses on addressing past trauma and preventing future trauma (Substance Abuse and Mental Health Services Administration [SAMHSA], 2023). Trauma-informed care has been implemented in child-serving systems, including child welfare and juvenile justice (Bargeman et al., 2021; Zhang et al., 2021), and is increasingly adopted in educational settings (Thomas et al., 2019). Research indicates that trauma-informed care in early childhood settings positively influences children’s behavior. For example, Tucker et al. (2021) conducted a randomized controlled trial of a trauma-informed intervention in preschool classrooms and found that children in the intervention group increased their prosocial behavior in attachment, self-regulation, problem-solving, and making friends. Furthermore, trauma-informed care has been shown to enhance early childhood teachers’ self-care strategies, communication with children and families, and classroom management (Douglass et al., 2021; Tucker et al., 2017; Whitaker et al., 2019), while also reducing the likelihood of using expulsion as a disciplinary measure (Loomis et al., 2023; Loomis & Panlilio, 2022). Although there are various definitions and frameworks for trauma-informed care, many scholars agree on two key components: trauma-informed attitudes and practices.

### Trauma-Informed Attitudes

Trauma-informed attitudes refer to the belief in the importance of using trauma-informed care to meet the needs of children who have experienced trauma (Baker et al., 2016). Trauma-informed attitudes are considered a key component of trauma-informed care because they influence perceptions and behaviors. In the classroom, trauma-informed attitudes can affect how a child’s behavior is interpreted and addressed (Baker et al., 2021; Loomis et al., 2023). For example, a teacher with lower trauma-informed attitudes may perceive behavior as deceitful and manipulative, whereas a teacher with higher trauma-informed attitudes may interpret a child’s behavior as a coping mechanism (Baker et al., 2021). Higher trauma-informed attitudes among teachers are also linked to a lower risk of expulsion for children (Loomis et al., 2023) and to decreased feelings of burnout and stress among teachers (Minne & Gorelik, 2022).

### Trauma-Informed Practices

Trauma-informed practices are strategies and supports that are used to support children who have experienced trauma. There is no single checklist of trauma-informed practices in education; instead, a variety of practices can be considered trauma-informed (Thomas et al., 2019). However, the ultimate goal of these practices is to build relationships and create supportive, predictable classroom environments (Chudzik et al., 2023). Some frameworks for trauma-informed practices in education emphasize underlying principles, such as connection, flexibility, and predictability (Venet, 2021). Others concentrate on specific strategies, including establishing clear expectations for safety, prioritizing relationships, fostering a sense of belonging, and teaching self-regulation skills (Alexander, 2019).

### Purpose of the Study

Previous research has examined the trauma-informed attitudes of preschool teachers (Guerrero et al., 2022; Loomis et al., 2023; Loomis & Felt, 2021), yet fewer studies have investigated trauma-informed practices (Chudzik et al., 2023). Additionally, it is essential to explore trauma-informed attitudes and practices together, as these may not always align (Herman & Whitaker, 2020). Furthermore, ECSE teachers are often excluded from the research, despite the increased risk of children with disabilities experiencing trauma (Chudzik et al., 2023). Therefore, the purpose of this study was to explore the trauma-informed attitudes and practices of ECSE teachers and understand their use of trauma-informed care. The research question for this study was: What are ECSE teachers’ trauma-informed attitudes and practices?

### Theoretical Frameworks

This study is grounded in trauma theory. Trauma theory is built on the notion that individuals, including children, are impacted by traumatic experiences (Alisic et al., 2011; Goodman, 2017; Herman, 1997). This theory calls for both an understanding of the role that experiencing trauma has on an individual and the use of this understanding to deliver services and supports in a trauma-informed way (Harris & Fallot, 2001). Additionally, trauma theory rejects the notion that behavior is a result of a deficit or as something that needs to be fixed and instead views behavior as a coping mechanism or adaptation to experiencing trauma (Goodman, 2017; Herman, 1997). Applying trauma theory in education settings requires teachers to recognize how trauma impacts children’s development and behavior in the classroom. In turn, this recognition of the impacts of trauma requires the use of trauma-informed care. While trauma-informed care does not necessarily mean educators directly addressing trauma, it does signify the need to conceptualize the child’s presentation and behavior through the lens of trauma and the need to implement trauma-informed practices. We used this theory to guide the design, implementation, and analysis of this study.

## Methods

**Fig. 1:**
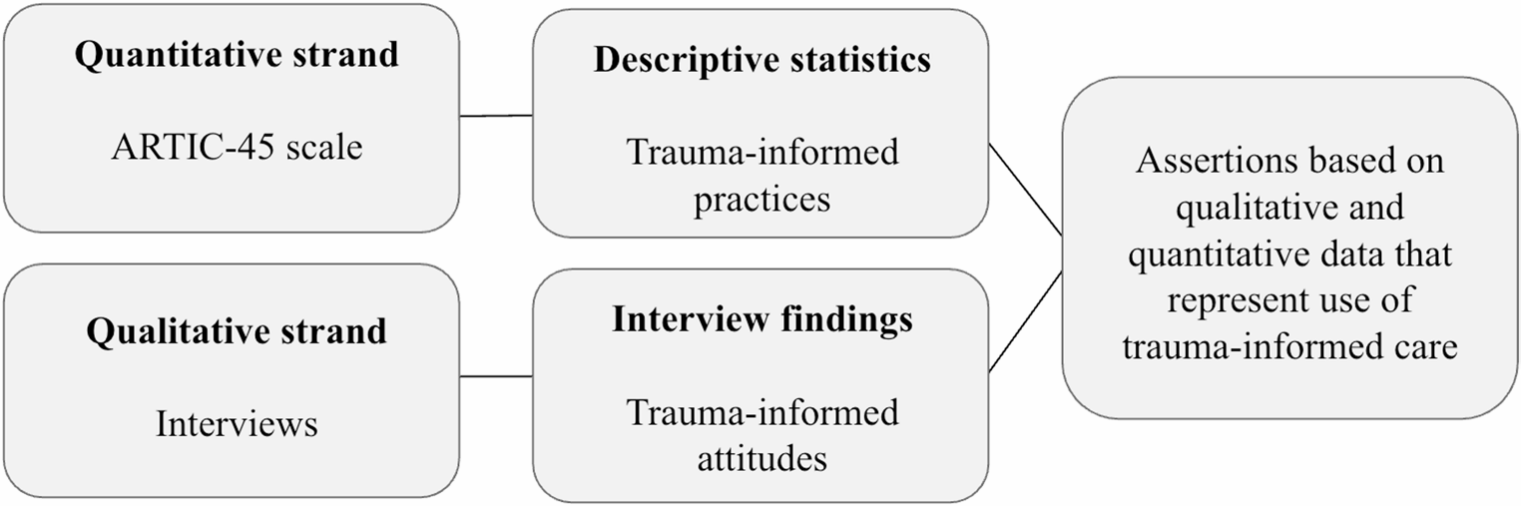
Mixed methods research design

This study followed a convergent mixed methods design (Greene, 2007). Quantitative data and qualitative data were collected separately and then merged for interpretation (see Fig. 1 for an overview of the study design). Quantitative data (i.e., survey data) was collected to measure trauma-informed attitudes, and qualitative data (i.e., interviews) was collected to explore trauma-informed practices. The purposes for mixing methods were threefold: (a) development, as we used survey constructs to design interview questions, (b) complementarity, as we looked for areas of similarity in the quantitative and qualitative data, and (c) initiation, as we looked for areas of divergence across the data sources. We piloted the study with three participants who provided feedback. This research study was approved by our university’s institutional research board in Summer 2023.

### Positionality

The research team consisted of three ECSE researchers with an interest in trauma and trauma-informed care. All three authors have experience working with children with disabilities in the classroom and identify as white women, which informed their decision making during the design of this study and the analysis of the data. The primary researcher’s experience as an ECSE teacher and as an advocate for trauma-informed care had the most significant influence on the design of this study and the interpretation of the data. Additionally, our perceptions of trauma and trauma-informed care impacted how data was interpreted, and these interpretations may be different from those of participants of different backgrounds. Moreover, as mixed methods researchers and in line with the purpose of the study, we were explicitly looking for areas of similarity and difference in the qualitative and quantitative data.

### Recruitment and Participants

Participants were identified through purposeful sampling (Patton, 2015). To participate in the study, individuals had to be ECSE teachers working in a public preschool program. The first author contacted administrators of public preschools in a Midwestern state and requested they share information about the study with their teachers. Interested individuals were encouraged to contact the primary researcher. Twelve ECSE teachers took part in this study. We used an all-sample sampling approach, where all participants contributed to both the quantitative and qualitative components of the study; this allowed for a thorough exploration by integrating quantitative and qualitative data for each participant (Teddlie & Tashakkori, 2009). All participants identified as white women. They averaged 16 years of teaching experience, although there was a wide range, with one participant in her first year of teaching and another in her 35th year. Additionally, participants came from urban, suburban, and rural parts of the state. All participants taught either full-day or half-day preschool in public settings with children who had disabilities, developmental delays, and/or who were considered at-risk for a disability. On average, participants had 20 children in their class, ranging from 19 to 35. See Table 1 for participant demographics.

**Table 1:** Participant demographics

Participant	Race/Ethnicity	Gender	Years Teaching	Highest Degree	Type of Program	Part of state
Ava	White	Female	5	Bachelor’s	Full day	Urban
Beatrice	White	Female	14	Bachelor’s	Full day	Urban
Cora	White	Female	20	Master’s	Full day	Rural
Demi	White	Female	26	Master’s	Half day	Suburban
Flora	White	Female	1	Bachelor’s	Full day	Urban
Gina	White	Female	7	Master’s	Half day	Suburban
Harper	White	Female	12	Bachelor’s	Full day	Urban
Inez	White	Female	8	Bachelor’s	Half day	Urban
Jade	White	Female	27	Bachelor’s	Full day	Urban
Kayla	White	Female	35	Master’s	Full day	Suburban
Leah	White	Female	20	Master’s	Half day	Rural
Maria	White	Female	20	Bachelor’s	Half day	Urban

### Data Collection

We collected data in two phases: quantitative data in phase one and qualitative data in phase two. The first author collected all data.

#### Phase 1: Survey Data Collection

First, participants completed an online survey using Qualtrics that included demographic questions and the Attitudes Related to Trauma-Informed Care (ARTIC-45) scale (Baker et al., 2016). The ARTIC-45 is a 45-item Likert scale developed by the Traumatic Stress Institute and is psychometrically validated with educators to measure trauma-informed attitudes. The ARTIC scale has demonstrated factor structure, internal reliability, and temporal consistency (Baker et al., 2016), along with construct validity (Baker et al., 2021), and has a Cronbach’s alpha of 0.93. The survey consists of seven subscales: (1) underlying causes of problem behavior/symptoms, (2) responses to problem behavior, (3) on-the-job behavior, (4) self-efficacy, (5) reactions to the work, (6) personal support of trauma-informed care, and (7) system-wide support of trauma-informed care. Each subscale includes items that reflect participants’ personal beliefs regarding trauma-informed attitudes and care. For example, participants rated on a scale of 1–7 which of the following two statements aligned more closely with their personal belief: “focusing on developing healthy, healing relationships is the best approach when working with students with trauma histories,” (1) and “rules and consequences are the best approach when working with students with trauma histories” (7). The ARTIC scores directly assess participants’ overall attitudes, where higher ARTIC scores indicate more positive trauma-informed attitudes (Baker et al., 2016).

#### Phase 2: Interview Data Collection

Then, participants participated in two semi-structured interviews, based on Seidman’s (2013) interviewing structure, which aims to have participants reconstruct their experience of the topic of study in an in-depth way, primarily through the use of open-ended interview questions. The interview protocols were developed based on the ARTIC survey questions, components of SAMHSA’s trauma-informed care framework, and what we wanted to know about participants’ use of trauma. Both interviews were conducted on Zoom and audio-recorded with participant consent. The first interview focused on building rapport and gathering context about each participant (e.g., information about their school, their classroom, their teaching practices, and their training) and lasted an average of 37 min (range 25–47). The second interview focused on the details of participants’ use of and understanding of trauma-informed practices (i.e., their definition of it, practices they consider trauma-informed). This interview took an average of 64 min (range 35–77).

### Data Analysis

#### Quantitative Analysis

The first author used descriptive statistics to analyze the quantitative data. First, data was downloaded from Qualtrics and imported into Microsoft Excel for analysis using the scoring spreadsheet provided by the Traumatic Stress Institute, the creators of the scale. Data was checked for missingness. Then, each participant’s ARTIC score was determined by calculating the mean for the composite score. This was also done for each of the seven ARTIC subscales.

#### Qualitative Analysis

Qualitative data was analyzed using the iterative three steps outlined by Miles et al. (2020). This method of analysis allowed for a detailed account of the data through a flexible approach. The first and second authors analyzed the data using MaxQDA, a qualitative analysis software. First, the interviews were sent for professional transcription and the first author listened to each interview recording while reading the transcripts, checking for accuracy. Then, the first and second authors coded the interview data using deductive and inductive coding, capturing data pertinent to the research questions (Saldana, 2021). Each transcript was read and coded independently, and then we came together to discuss any differences in coding and come to a consensus. We repeated this process until each interview was coded. After the first round of coding, we used pattern coding to identify similarities in codes to group them into categories (Saldana, 2021). The final step in the qualitative analysis was to draw conclusions. The first author created tentative findings and verified them by returning to the data and looking for data to confirm or disconfirm them. Then, the findings and accompanying evidence were shared with the second author as a form of peer debriefing. She provided feedback, which was discussed and incorporated to form the final conclusions.

### Mixed Methods Analysis

After the separate analysis of the quantitative and qualitative data, we analyzed the data together. We integrated the data by merging the quantitative and qualitative results to compare and generate a more complete understanding of participants’ use of trauma-informed care (Creswell & Plano Clark, 2017). The first author created joint data displays that combined the survey and interview data (Guetterman et al., 2015). This mixed methods integration was used to form meta-inferences and final assertions that answered the research questions (Tashakkori & Teddlie, 2008). The first author developed five assertions that summarized the mixed data and answered the research questions. First, evidence from survey and interview data were reviewed and combined. Then, the assertions were shared with the second author, who reviewed the assertions and evidence to confirm, disconfirm, and/or clarify the initial assertions. This resulted in minor edits to the assertions, creating the five final assertions addressed in the findings.

### Mixed Methods Quality

Multiple steps were taken to ensure the quality of this mixed methods study (Leko et al., 2023). To ensure strong component designs, we adhered to the quality indicators for the chosen methods. We conducted member checks with participants, engaged in peer debriefing, and used a collaborative data analysis process to ensure trustworthiness with the qualitative data (Brantlinger et al., 2005; Tracy, 2010). For the survey, we chose a measure that was psychometrically validated with the population we were working with (i.e., educators) and that adequately covered the topic of trauma-informed attitudes. Additionally, our sample integration (i.e., all participants completed both the quantitative and qualitative portions) meant that we could draw conclusions from all participants.

## Findings

**Fig. 2:**
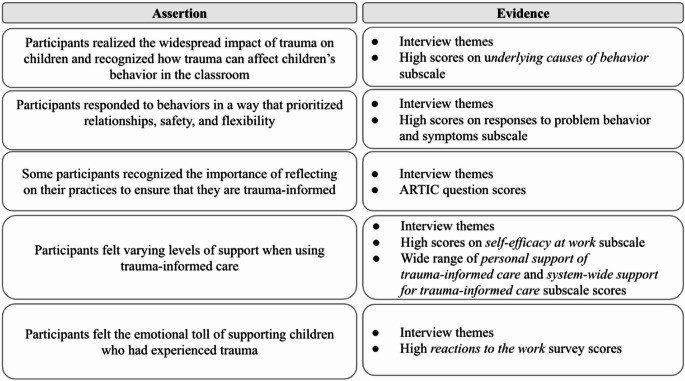
Mixed methods assertions

First, we provide a brief overview of participants’ ARTIC scores. Then, we present the assertions, which are based on the quantitative and qualitative data mixed together. See Fig. 2 for an overview of the assertions.

### ARTIC Scores

**Fig. 3:**
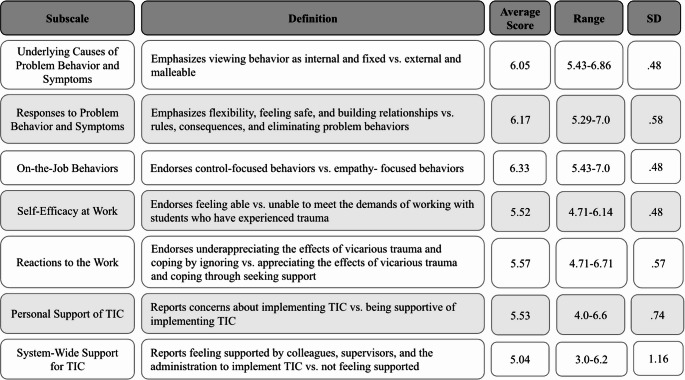
Participant ARTIC scores. Note. Definitions adapted from Baker et al. (2016). Score of 1 = low trauma informed attitudes, score of 7 = high trauma-informed attitudes

Participants had an average overall ARTIC score of 5.7 (range 5.29–6.44, *SD* = 0.38), reflecting relatively high trauma-informed attitudes. Regarding the ARTIC subscales, average scores varied, with the highest score of 6.14 in the *Responses to Problem Behavior and Symptoms* subscale, and the lowest score was 5.04 in *System-Wide Support for Trauma-Informed Care*. See Fig. 3 for the average scores of all ARTIC subscales.

### Mixed Methods Assertions

#### Participants Realized the Widespread Impact of Trauma on Children and Recognized How Trauma Can Affect Children’s Behavior in the Classroom

Participants demonstrated an understanding of how trauma impacts children’s behavior in the survey and interviews. The ARTIC subscale *Underlying Causes of Behavior* evaluates whether participants perceive behavior as intentional and fixed or as symptoms of past experiences. The average score for this subscale was 6.05 out of 7 (range = 5.43–6.86), indicating that participants largely viewed children’s behavior as a symptom of their past experiences. For instance, Flora, who scored 6.29 on this subscale, described a child she had difficulty with due to his behavior. She shared:

He has a really hard time sitting and focusing on what we’re doing and even listening, but mom is about eight months pregnant, and his parents fight a lot, so I think a lot of it could be attention-seeking because he just doesn’t get that at home, which I totally understand.

In addition to recognizing the impact of trauma on behavior, many participants described how trauma-informed care enables them to explore children’s behaviors more deeply. They looked beyond surface-level behaviors and shifted their perspective on how behavior is understood. Maria, who scored 6.43 on the *Underlying Causes of Behavior* subscale, shared, “Their behavior may not be a direct impact of something that I’m doing. It could be maybe something I’ve triggered…having that greater understanding of trauma has helped me to not just look at what the outside behavior is telling me.” This quote illustrates complementarity in the mixed methods data, as Maria’s higher score on the *Underlying Causes of Behavior* subscale aligns with her in-depth description of how her views on behavior shifted after learning more about trauma. This pattern was seen across participants; many repeatedly noted that utilizing trauma-informed care changed their perceptions of children’s behavior.

**Fig. 4:**
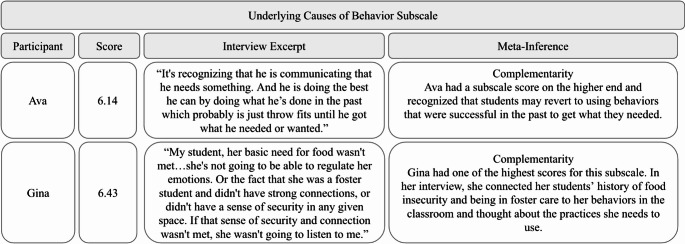
Underlying causes of behavior joint display. Note. Scores were on a scale of 1-7, where 1 = less trauma-informed approach and 7 = more trauma-informed approach. Average score for the Underlying Causes of Behavior subscale was 6.05 (range = 5.43-6.86, SD = .48)

Overall, the interview data and the *Underlying Causes of Behavior* subscale showed complementarity in participants’ understanding of the impact of trauma. Additionally, participants recognized the impact that trauma can have on children’s behavior in the classroom. See Fig. 4 for a joint display of the *Underlying Causes of Behavior* subscale scores, accompanying interview excerpts, and meta-inferences.

#### Participants Responded To Behaviors in a Way that Prioritized Relationships, Safety, and Flexibility

Participants endorsed the importance of relationships, safety, and flexibility in their ARTIC data. The subscale *Responses to Problem Behavior and Symptoms* evaluates whether participants prioritize flexibility, feeling safe, and building healthy relationships over rules and consequences when responding to students who have experienced trauma. The average score for this subscale was 6.17 (range 5.29–7.00.29.00), indicating that participants largely prioritized flexibility, feeling safe, and building healthy relationships over rules and consequences when supporting students who have experienced trauma. Similarly, the subscale *On-The-Job Behavior* examines whether participants support empathy-focused behaviors or control-focused behaviors when supporting students who have experienced trauma. The average score for this subscale was 6.33 (range 5.43–7.00.43.00), suggesting that participants largely endorsed empathy-focused behaviors. Overall, this data demonstrates that participants valued responding to behavior in ways that prioritize empathy, relationships, and flexibility.

In the interviews, participants also shared practices focused on relationships, safety, and flexibility. For example, Gina, who scored 6.00 in both the *Responses to Problem Behavior and Symptoms* and *On-The-Job Behavior* subscales, reflected on one child in her class who pushed her to think more about trauma-informed care:

I had my student who was a foster student…it really pushed me towards more flexible thinking. So like an expectation is not the end all be all. So, she wasn’t sitting at group time with everybody else. But she was sitting at the table eating her snack and listening to group time. It does not have to be black and white like that.

Gina had high ARTIC scores that endorsed the importance of flexibility, and shared how she practiced flexibility by changing how group time looks depending on students’ needs, indicating complementarity in the data. In contrast, Cora had ARTIC scores of 5.29 on the *Responses to Problem Behavior and Symptoms* subscale and 5.43 on the *On-The-Job Behavior* subscale, which was the lowest among the participants. However, she shared how she prioritized helping children feel safe in the classroom, stating, “We do a lot of talking about school is safe. Which is something that I think for a lot of kids who have had some kinds of trauma they really need to know that they are safe at school.” Despite having the lowest ARTIC scores for these subscales, Cora still recognized the importance of safety in the classroom, indicating divergence between the survey and interview data.

**Fig. 5:**
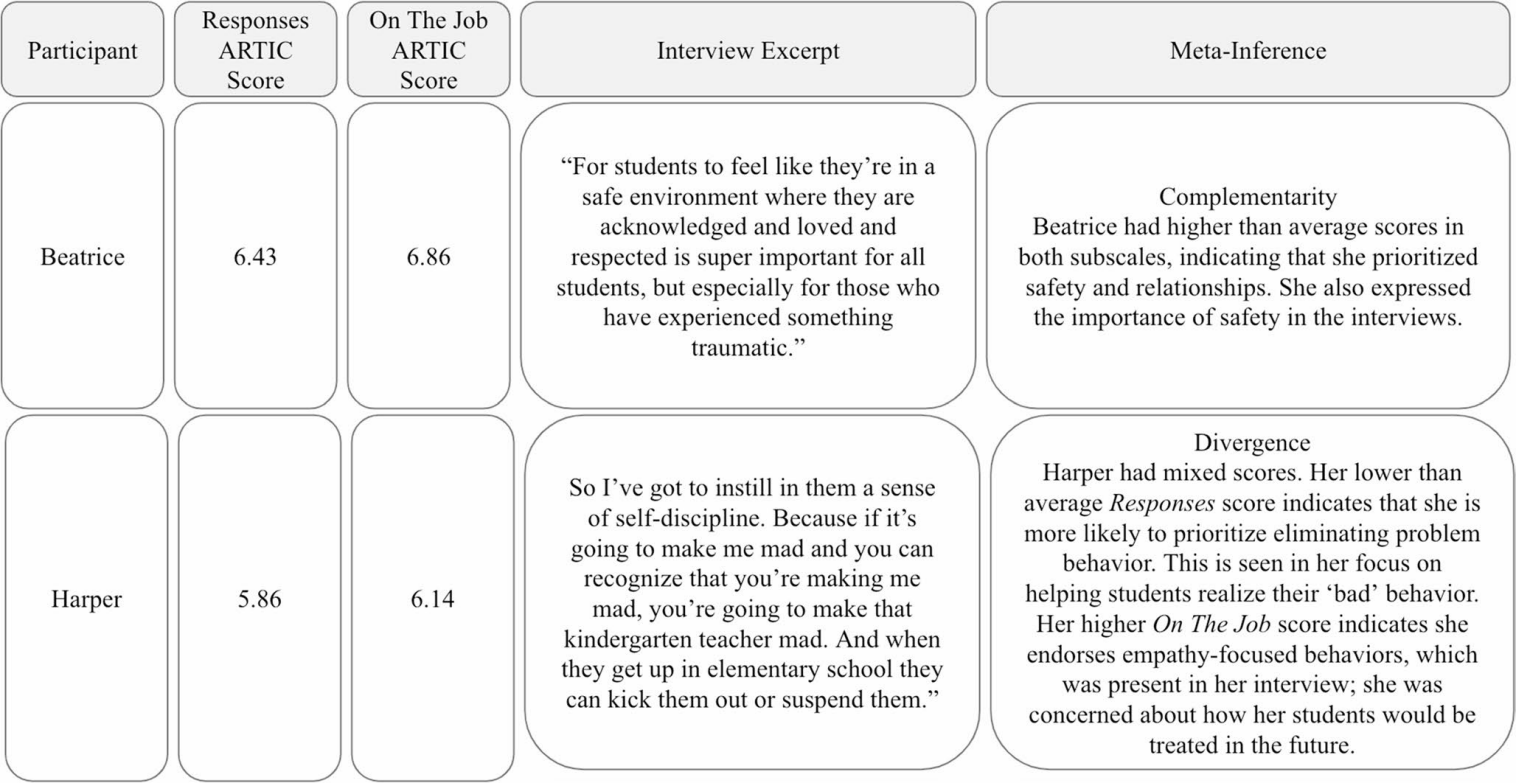
Responses and on-the-job joint display. Note. Scores were on a scale of 1-7, where 1 = less trauma-informed approach and 7 = more trauma-informed approach. Average score for the Responses to Behavior and Symptoms subscale was 6.17 (range = 5.29-7.0, SD = .58). Average score for the On the Job Behavior subscale was 6.33 (range = 5.43-7.0, SD = .48

Overall, based on interview and survey data, participants shared practices that prioritize relationships, safety, and flexibility, regardless of ARTIC score. Some participants also recognized the importance of reflecting on their practices, ultimately helping them to become more trauma-informed. See Fig. 5 for a joint display that includes *Responses to Behavior* and *On-the-Job Behavior* subscale scores, along with interview excerpts and meta-inferences.

#### Some Participants Recognized the Importance of Reflecting on Their Practices to Ensure That They are Trauma-Informed

For trauma-informed care to be successful, teachers must critically examine their practices to ensure they are effectively supporting children who have experienced trauma and be open to making changes when their practices are lacking. This openness to reflection and adaptation was evaluated using the ARTIC scale. One question on the ARTIC was, “If things aren’t going well, it is because the students are not doing what they need to do,” versus “If things aren’t going well, it is because I need to shift what I’m doing.” The average score was 6.25 (range 5.00–7.00), suggesting that participants were likely to shift their practices to better support children. Not all participants mentioned reflecting on their practices in the interviews, but those who did rated this question a 6 or 7. For instance, Beatrice, who scored a 7.0 on this question, shared how she used to require the students in her class to sit in a certain way on the carpet in the classroom, but after learning more about trauma-informed care, she recognized the importance of flexibility and allowing students to sit in a manner that is comfortable for them. She reflected on her practice and acknowledged it might not be supportive for children who have experienced trauma. Consequently, she modified that practice to become more trauma-informed by letting children choose how they sit on the carpet. By doing this, children gained more agency and control over themselves, making it more trauma-informed. Beatrice’s high ARTIC score for this survey question and her interview data about changing practices indicate complementarity. Similarly, Demi, who also scored 7.0 on the ARTIC, talked about a transformation in her classroom management as she adopted a more trauma-informed approach. She described shifting from a rigid and inflexible style to one that was more trauma-informed, saying:

I was really good at classroom management. That was something that I really prided myself on…I definitely wasn’t looking for meaning behind behavior. But now, really understanding that their behavior has meaning, and looking not at it as a personal attack or a challenge, but as meaning, they’re trying to tell me something with this behavior, and when I find out what that is, then I’ll know how to help them better.

By being able and willing to critically examine and reflect on their practices and policies, participants like Beatrice, Demi, and others recognized how they were not supporting children who have experienced trauma, and then adjusted their practice to be more trauma-informed.

### Participants Felt Varying Levels of Support when Using Trauma-Informed Care

ARTIC data indicated that participants experienced varying levels of support regarding the use of trauma-informed care. Several subscales contributed to assessing the levels of support for implementing trauma-informed care, including *Self Efficacy at Work* (average score = 5.52), *Personal Support of Trauma-Informed Care* (average score = 5.53), and *System-Wide Support for Trauma-Informed Care* (average score = 5.04). These subscale scores were lower than those measuring understanding of and responses to trauma, potentially suggesting that participants had the knowledge and skills required to implement trauma-informed care but lacked the necessary support to do so effectively. In interviews, participants echoed the varying levels of support they felt when using trauma-informed care, which was represented in the survey data through the supports and barriers they encountered. Factors influencing participants’ feelings of support in using trauma-informed care included their administrators, colleagues, and school resources.

**Administrators.** Some participants felt supported by their administrators, which helped them implement trauma-informed care. For example, when asked about support for using trauma-informed care, Ava shared, “They [administrators] would support any learning opportunities and do what they could to get me a sub.” However, others, like Kayla, did not feel supported by their administrators. She expressed disappointment, feeling that administrators placed too much on her plate, making it difficult to use trauma-informed care. She shared, “Administration goes, ‘Oh, we’ve got to do all this stuff,’ so they throw it up, and then it’s like, okay, this is way too much for me to do.” This variety in administrative support was also reflected in the survey data. One ARTIC question asked *There is not much support from the administration for my work* (1) vs. *There is clear indication that the administration supports my work* (7). The average score for this question was 5.33, with a range of 3.0–7.0, indicating a variety of experiences with administration regarding support for trauma-informed care use.

**Colleagues.** Despite varying levels of support from their administration, most participants expressed feeling supported by their colleagues in implementing trauma-informed care. They mentioned being able to approach their colleagues to vent, discuss problems, and gain perspectives on various situations. This support came from classroom aides, teachers from different classrooms, and related service providers such as school social workers and family support staff. For instance, Cora highlighted the advantages of having family support staff, stating, “We have two family support workers…I can reach out and just say, hey, this was mentioned to me at conferences, do you want to see if you can follow up or what do you have that I can send home?” These participants acknowledged that they could not effectively implement trauma-informed care alone; they needed other staff members to be trauma-informed as well.

**School Resources.** Other participants discussed school resources, or the lack thereof, that affected their ability to use trauma-informed care. These issues included insufficient staff and limited opportunities for professional development in trauma-informed care. For example, Inez described how a lack of trauma-informed classroom aides hinders her ability to use trauma-informed care. She shared, “We don’t have enough educated people…our district is so desperate that they’re hiring bodies. They’re not hiring people that know anything about children…So when you have a para in your room that doesn’t know anything that adds to the stress.” Additionally, some participants reflected on the lack of in-school mental health supports that are available for children who have experienced trauma. Inez shared that there are no counselors available for students at her school, saying “We need counselors in the schools. We don’t have that in early childhood.” On the other hand, Demi shared that she has access to social workers at her school but that they do not provide direct support to students. She shared why this was a barrier, saying:

For kids that you know there has been trauma… I just feel like we are just in survival mode trying to keep everybody safe, and if we had direct service minutes with social workers, I think that would help them start to move forward instead of just being in the status quo.

**Fig. 6:**
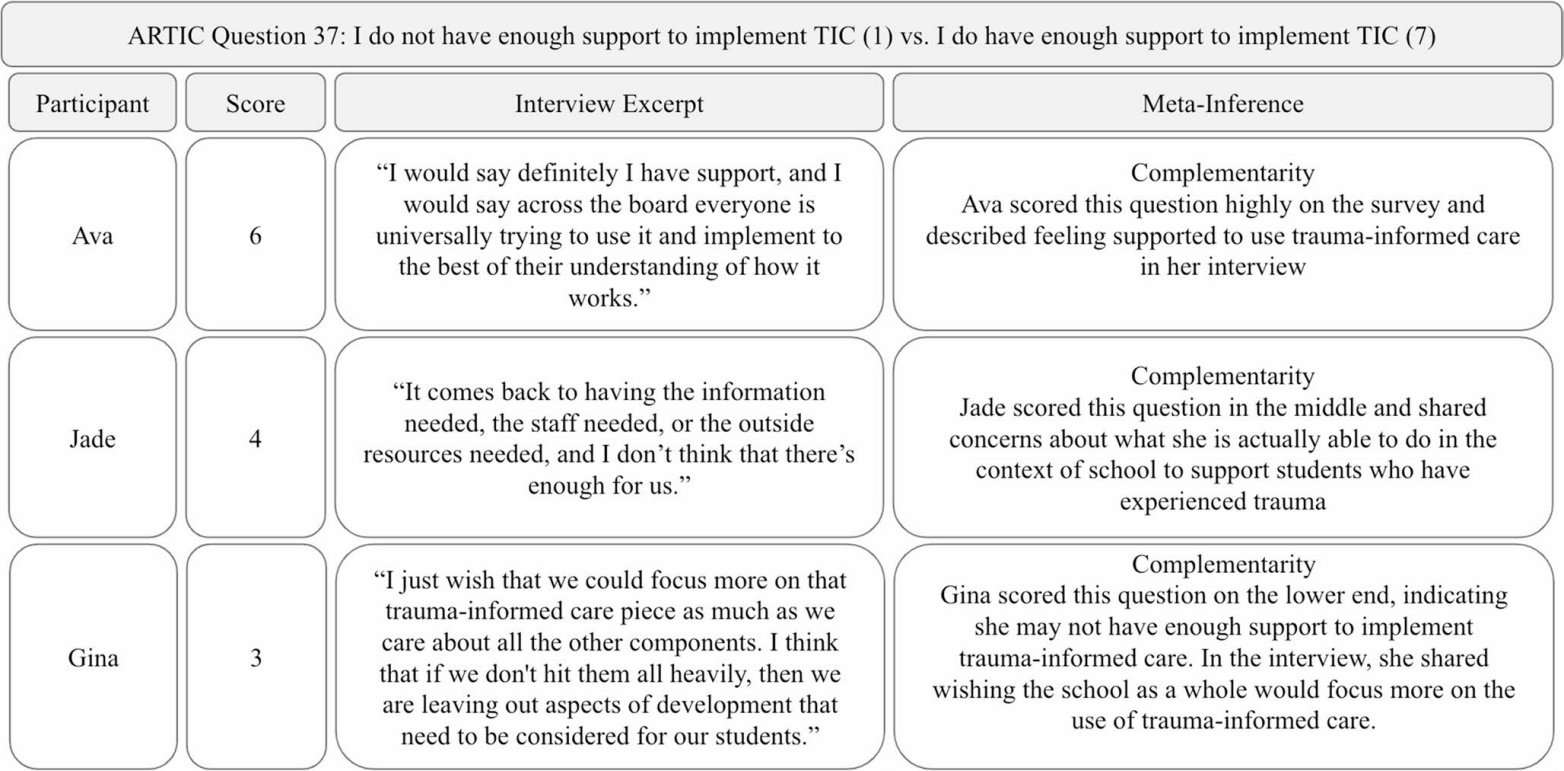
Feelings of support joint display. Note. Scores were on a scale of 1-7, where 1 = less trauma-informed and 7 = more trauma-informed. The average score for this question was 4.91 (range = 1-6)

Overall, participants expressed mixed feelings about the support they received for using trauma-informed care. See Fig. 6 for a joint display that includes a survey question regarding feelings of support, along with relevant interview excerpts and meta-inferences.

#### Participants Felt the Emotional Toll of Supporting Children who Had Experienced Trauma

In the interviews, participants discussed the emotional toll that using trauma-informed care had on them. For some, using trauma-informed care evoked both positive and negative feelings, such as “rewarding,” “frustrating,” and “tiring.” Flora reflected on the positive emotions she experienced when successfully supporting a child using trauma-informed care, saying, “It’s a good feeling when you are able to give them the help they need. Like okay, I am helping, and then you see it slowly making a difference. So it’s like a feeling of joy.” Other participants highlighted the negative emotions they encountered. Maria explained how she sometimes felt during moments of supporting a child who experienced trauma, stating, “I feel stressed because I am in that fight or flight. I want to make sure that the environment is safe. Sometimes I’m on the verge of tears.” For Maria, using trauma-informed care meant being hypervigilant and concerned about the classroom environment, often leading to her stress.

Many participants recognized the importance of their well-being in successfully implementing trauma-informed care. Ava summed this up by saying, “There has to be some kind of balance. Like you have to take care of yourself and your needs before you can take care of anyone else’s needs.” This sentiment was also reflected in the ARTIC data. One question asks, “*How I am doing personally is unrelated to whether I can help my students* (1) vs. *I have to take care of myself personally in order to take care of my students* (7).” The average score for this question was 6.16 (range 2.0–7.0), indicating that most participants agreed on the importance of taking care of themselves in order to care for their students. Notably, Ava was the one participant who scored this question as a 2.0 despite expressing in the interview the importance of self-care, indicating divergence. However, participants also discussed the barriers they encounter in taking care of themselves, such as bringing work home or not having time for self-care. Demi described this difficulty saying:

It’s overwhelming, and it’s like emotionally depleting for myself for sure. Like those are hard times, and you can’t just like self-care, whatever, like it’s not good. Leave it at school, like none of that happens. We all know that. So, it’s very emotionally depleting.

Inez also described the impact that teaching children who have experienced trauma has had on her, saying “I try to leave my problems from home at home. I try to be positive, but I’m human, so once in a while it does get in the way. We’re tired, and we’re worn out, and we have breaking points.” Overall, many participants were aware of the importance of taking care of themselves in order to support the students in their class through trauma-informed care.

**Fig. 7:**
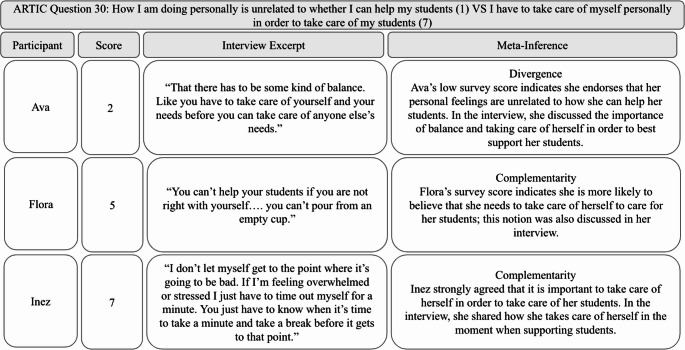
Note. This data is mandatory. Please provide

In summary, participants acknowledged how supporting children who have experienced trauma and using trauma-informed care impacts them both negatively and positively. They recognized the importance of self-care for effectively supporting their students; this insight was evident in both the interview and survey data. See Fig. 7 for a joint display that includes a survey question on well-being, relevant interview excerpts, and meta-inferences.

## Discussion

In this study, we aimed to understand ECSE teachers’ use of trauma-informed care by exploring their trauma-informed attitudes and practices. Overall, we found that participants had relatively high trauma-informed attitudes and shared a variety of experiences with trauma-informed practices, some of which did not align with their attitudes score. Next, we discuss key findings, implications, and directions for future research.

First, the participants in this study reported feeling confident in their ability to use trauma-informed care. However, many participants faced barriers that prevented them from using trauma-informed care in the way they intended. Some of these barriers were individual, such as an administrator’s disinterest in trauma-informed practices, whereas others were systemic issues, such as a staff shortage that made it difficult to adequately provide individual support for children. It is not surprising that participants reported this lack of support as a barrier, given that program-wide buy-in is a key component to successful trauma-informed care use (SAMHSA, 2023; Wassink-de Stigter et al., 2022). This finding has implications for research and practice. When considering professional development opportunities related to trauma-informed care, all staff members need to be included, encompassing related service providers, administrators, and classroom aides, and teachers. Moreover, future research can explore administrators’ perspectives on supporting children who have experienced trauma and trauma-informed care as they are a key driver of trauma-informed care implementation (Wassink-de Stigter et al., 2022).

Second, this study points to disparities between trauma-informed attitudes and practices. There were instances where participants had lower trauma-informed attitudes and shared trauma-informed classroom practices. This mismatch between attitudes and practices has been discussed in both the context of trauma-informed care (Chudzik et al., 2022; Whitaker & Herman, 2020) and in the broader context of human behavior (Ajzen, 1991; Kroesen et al., 2017). This has implications for both research and practice. When considering how ECSE teachers use trauma-informed care, it is necessary to rely on more than just trauma-informed attitudes, as doing so could lead to a misleading perception of teachers’ use of trauma-informed care, either underestimating or overestimating their trauma-informed practices. Much of the literature on trauma-informed care in education relies on quantitative methods (Chudzik et al., 2023; Thomas et al., 2019). Assessment tools such as the ARTIC are often used to measure trauma-informed care. However, it is difficult to determine how quantitative trauma-informed attitudes translate to actual trauma-informed classroom practices ​​(Orapallo et al., 2021; Loomis et al., 2023). This highlights the need for multiple evaluative and research methods to be used to more accurately capture the use of trauma-informed care.

Finally, it is important to consider how to strengthen the use of trauma-informed care in ECSE teachers. Prior research has demonstrated an increase in trauma-informed attitudes after training (Bartlett & Rushovich, 2018; Loomis et al., 2023). However, this research has focused on changes in attitudes rather than practices, underscoring the need for future research in this area. Future research can explore ways of changing trauma-informed practices through professional development and other types of training such as reflective supervision. Reflective supervision is a relationship-focused supervisory process that supports provider reflection in a supportive, non-judgmental environment that aims to improve practitioners’ practice by learning to question their thoughts, feelings, and actions in situations (Huffhines et al., 2023; Tobin et al., 2024). While research has shown reflective supervision has increased early childhood professionals’ insightfulness into children’s behavior (Amini Virmani & Ontai, 2010), self-efficacy and ability to handle job-related stress (Frosch et al., 2018), and well-being (Huffhines et al., 2023), reflective supervision with the use of trauma-informed care is still in its early stages (Loomis et al., 2024). The positive impact that reflective supervision has on infant and early childhood mental health professionals in supporting children and families who have experienced trauma warrants investigation.

### Limitations

There are a few limitations to consider when interpreting the results of this study. First, all participants in this study were white women. This means this study is missing insights from teachers of color and male teachers who may have different experiences with trauma and trauma-informed care, which would impact their trauma-informed attitudes and practices. Future research can make more targeted efforts to recruit diverse ECSE teachers. Additionally, self-selection bias may have impacted this study, as those who chose to participate may be more informed about trauma and trauma-informed care, thereby having higher trauma-informed attitudes and using trauma-informed practices. Finally, participants’ survey scores may be inaccurate due to social desirability bias. Despite these limitations, the findings from this study add key information to the literature base about how teachers’ trauma-informed attitudes and practices come together to implement trauma-informed care in ECSE.

## Conclusion

The current study explored trauma-informed attitudes and practices of ECSE teachers. We found areas of complementarity and divergence in the data, where trauma-informed practices and attitudes aligned or did not align. These findings address existing gaps in understanding how trauma-informed care is used in ECSE settings. These findings also highlight the complexities of trauma-informed attitudes and practices and the implications for research and practice because of this complexity. A deeper understanding of trauma-informed attitudes and practices, the two components of trauma-informed care, can lead to better ECSE environments for children with disabilities who have experienced trauma.

## Data Availability

De-identified data are available from the corresponding author upon reasonable request after publication.
